# DOPESLAM: High-Precision ROS-Based Semantic 3D SLAM in a Dynamic Environment

**DOI:** 10.3390/s23094364

**Published:** 2023-04-28

**Authors:** Jesse Roch, Jamil Fayyad, Homayoun Najjaran

**Affiliations:** 1 School of Engineering, The University of British Columbia, Kelowna, BC V1V 1V7, Canada; jroch@student.ubc.ca (J.R.); jfayyad@mail.ubc.ca (J.F.); 2 Faculty of Engineering and Computer Science, University of Victoria, Victoria, BC V8W 2Y2, Canada

**Keywords:** semantic SLAM, Deep Object Pose Estimation, Real-Time Appearance-Based Mapping, precision enhancement, object recognition

## Abstract

Recent advancements in deep learning techniques have accelerated the growth of robotic vision systems. One way this technology can be applied is to use a mobile robot to automatically generate a 3D map and identify objects within it. This paper addresses the important challenge of labeling objects and generating 3D maps in a dynamic environment. It explores a solution to this problem by combining Deep Object Pose Estimation (DOPE) with Real-Time Appearance-Based Mapping (RTAB-Map) through means of loose-coupled parallel fusion. DOPE’s abilities are enhanced by leveraging its belief map system to filter uncertain key points, which increases precision to ensure that only the best object labels end up on the map. Additionally, DOPE’s pipeline is modified to enable shape-based object recognition using depth maps, allowing it to identify objects in complete darkness. Three experiments are performed to find the ideal training dataset, quantify the increased precision, and evaluate the overall performance of the system. The results show that the proposed solution outperforms existing methods in most intended scenarios, such as in unilluminated scenes. The proposed key point filtering technique has demonstrated an improvement in the average inference speed, achieving a speedup of 2.6× and improving the average distance to the ground truth compared to the original DOPE algorithm.

## 1. Introduction

A common task required of autonomous mobile robots is the ability to generate a semantic map of the environment it is navigating through. The map itself can be created through means of simultaneous localization and mapping (SLAM), which can be either LiDAR-based [1,2], visual-based [3,4], or both [5,6]. While there are many approaches towards implementing SLAM, certain methods make it easier than others to semantically label objects found within the map. Visual SLAM approaches allow for the convenient evaluation of labeled objects by inspection, as the pixel color can be matched with the detected object. The advantage of LiDAR SLAM, however, is that because LiDAR has better ranging accuracy, the localization coordinates are more reliable than visual SLAM on its own [7]. This highlights the significance of using a combination of LiDAR and visual SLAM to retrieve accurate localization coordinates while retrieving point cloud RGB values, which are useful for object labeling. To complement SLAM in creating a semantic map, a reliable object detection algorithm is necessary. While many algorithms exist, there are several important qualities to consider, such as pose estimation, computation cost, and detection range. Because the map is a 3D representation of the surroundings, the pose of the identified objects is particularly important. While there have been advances in one-stage object detectors to include monocular depth estimation such as in [8], most algorithms that estimate depth are two-stage detectors, which have considerably increased computation times that can hinder real-time performance when tracking multiple classes [9,10]. However, real-time object labeling is not necessarily required if the goal is to achieve a high-accuracy semantic map and if it is not relied upon for navigation. There are several options available to combine semantic labels with mapping, though they come with vastly different implementation considerations. These methods include direct fusion as well as a loose-coupled or tight-coupled parallel fusion [11]. In direct fusion, the SLAM-generated 3D structure, which can take the form of a point cloud, mesh, or volumetric display, is used as the input to an object detection algorithm that outputs the annotated map. A constraint on this method is that the object detector must be able to accept a 3D structure as the input. Tight-coupled parallel fusion, on the other hand, extracts features from the environment, which are then used to identify objects and perform loop closure for mapping simultaneously. This has the benefit of using semantic information in a more significant role. While this approach has the potential to improve the robustness of SLAM, more research in this area is needed before it can be used effectively; great potential has been shown by methods that use a deep learning approach [11]. Finally, loose-coupled parallel fusion allows SLAM and object detection to be performed independently, and as such has no real-time requirements for implementation. This makes it convenient to perform SLAM during autonomous navigation, allowing it to be used for path planning in real-time, then the objects can be labeled in post-processing when more computation power is available. The semantic SLAM system presented in this paper, dubbed DOPESLAM, uses the Robot Operating System (ROS) to fuse Deep Object Pose Estimation (DOPE) [9] with Real-Time Appearance-Based Mapping (RTAB-Map) [5] through means of loose-coupled parallel fusion. The quality of DOPE’s depth estimation is evaluated experimentally while simultaneously determining the ideal size of a training dataset comprised of around 100,000 images. Furthermore, a method of precision enhancement through false positive reduction (FPR) is introduced, and a novel means of quantification is used to find the precision, recall, and F-score of a modified version of DOPE. The modification enables object recognition using a depth map as input, which allows for shape-based object recognition in darkness or other low-visibility conditions. FPR prioritizes precision by maintaining it between 99.5% and 85.7% while reducing the size of DOPE’s neural network from 86 to 21 layers, promoting an increase in frame rate from 8.6 Hz to 22.6 Hz. Additionally, FPR’s performance is compared with the performance of the original version while using the manually selected best detections as a baseline along with the worst detections to put DOPE’s effective range in perspective. Experimental results show that FPR achieves an average distance of 30.5 cm from the labeled objects to the ground truth, compared to 79.8 cm achieved by the original version, while the best detections had an average distance of 12.3 cm and the worst detections had an average distance of 184.8 cm.

In this paper, we present a solution to semantic SLAM systems; specifically, we propose a solution to tackle the crucial task of object labeling and 3D map generation in a dynamic environment. The main contributions of the paper are:

1. We propose an integration of Deep Object Pose Estimation (DOPE) and Real-Time Appearance-Based Mapping (RTAB-Map) through loose-coupled parallel fusion to generate and annotate 3D maps.
2. We present a solution that enhances the precision of the system by leveraging DOPE’s belief map system to filter out uncertain key points when predicting bounding box locations.
3. We propose an enhancement to the original DOPE algorithm to enable shape-based object recognition using depth maps, which can identify objects in complete darkness.
4. Finally, we apply a range of experiments to evaluate and quantify the overall performance of the proposed solution.

## 2. Related Work

There are many different implementations of semantic SLAM. One such approach is that of Kimera [2,12]. Kimera is a modular system that performs visual-based SLAM using up to four different modules to create a semantic map. It can use visual-inertial odometry (VIO), robust pose graph optimization (RPGO), meshing, and semantic modules. Because it has its own semantic module, there is no need to fuse it with an object recognition algorithm. While it is convenient to not require the use of LiDAR, its absence is somewhat noticeable in its performance. Kimera offers an average distance from object labels to the ground truth of between 35 and 48 cm during experiments [12]. This value is offered as a range, as it generates a mesh of the environment. Another attempt at semantic SLAM can be seen in [13]. This method restructures the semantic SLAM problem as an optimization problem involving the sensor states and landmark position estimates. It combines spatial positioning with semantic probabilities through data association. It uses simple landmarks with an estimate of the robot’s pose to navigate through various environments. As such, this is an example of an attempt at tight-coupled parallel fusion, with the semantic information used for localization. Experimental results showed that the estimated position of the landmarks was an average of 25 cm from the ground truth [13]. In [14], McCormac et. al proposed SemanticFusion, which is a method for building dense 3D semantic maps of indoor environments using a CNN-based approach. This approach takes as input a sequence of RGB-D frames from a handheld or mobile depth camera and outputs a dense 3D semantic map of the environment, with each voxel in the map labeled with a semantic category. The authors evaluated the SemanticFusion system on several datasets and demonstrated that it achieves state-of-the-art performance in terms of accuracy and efficiency. In [15], the authors proposed a method for incrementally building a model of a scene in real-time using both SLAM-based scene understanding and techniques for identifying objects and their positions within the scene. This approach uses a probabilistic method to predict which objects are in the scene at each new frame of video. The system then uses this information to incrementally build a model of the scene over time. In [16], Zheng et al. proposed a SLAM approach for robot-operated active understanding of unknown indoor scenes. Their algorithm is built on top of a volumetric depth fusion framework and performs real-time voxel-based semantic labeling over the online reconstructed volume. The authors showed through extensive evaluation that their method achieves efficient and accurate online scene parsing during exploratory scanning. Finally, in [17], the authors proposed an extension to a surfel-based mapping approach that integrates semantic information to facilitate the mapping process. Their algorithm deploys a fully convolutional neural network to extract important semantic features. Evaluations were carried out on the KITTI dataset with multiple static and dynamic objects.

## 3. System Components

**Figure 1:**
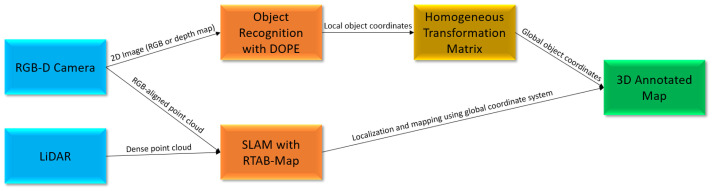
Flowchart illustrating the operation of the proposed DOPESLAM algorithm.

To implement the proposed system, there are several factors that need to be considered. First, DOPE needs to be trained to look for objects. After the model is trained to look for objects, the output coordinates provided by DOPE must be fused with the point cloud generated by RTAB-Map. The predictions from DOPE can then be further enhanced by implementing a false positive reduction (FPR) method. Furthermore, to enhance DOPE’s abilities and to support a novel method used to quantify precision, a modification allowing the use of depth maps as input for shape-based object recognition is introduced. Finally, the poses of all objects can be stored along with their locations relative to the 3D SLAM-generated map using a persistent state observation technique. This combination of elements is designed to produce a highly accurate 3D semantic map that is capable of operating in low visibility conditions. Figure 1 illustrates the overall architecture of the proposed algorithm.

### 3.1. Semantic Annotation

One of the most important abilities of an object recognition algorithm used to annotate a 3D map is the ability to accurately estimate the depth of the object. This is crucial in order to be able to place a label correctly within the scene. In addition to depth estimation, the range, frame rate, and simultaneous classes are important considerations; however, a fast frame rate and the ability to identify many objects at once are more important for a system designed to operate in real time. If real-time operation is not required, the focus can remain on depth estimation and detection range. These factors can immediately eliminate many algorithms when deciding on a suitable candidate for this application. While DOPE borders on the real-time operation and can identify multiple objects at once, its core strength lies in its depth estimation, and it has a reasonable detection range of approximately 150 cm. DOPE was trained using synthetic images generated by Nvidia’s Deep Learning Dataset Synthesizer (NDDS) [18], which is a plugin for Unreal Engine 4. It uses do-main randomization [19] and a 3D model to bridge the simulation-to-reality gap [20], and can generate any number of images. The images are varied in ways such as position, orientation, background, and lighting. NDDS introduces distraction objects to produce a robust dataset of labeled images that are perfect for training a model. NDDS can generate RGB, 8-bit, and 16-bit depth maps, as well as segmentation images, all of which can be useful for training a model. DOPE’s convolutional neural network (CNN) consists of 86 layers. It uses the first ten layers of VGG-19 [21] (trained on ImageNet) to generate the features used for object recognition. Following that, the features are dimensionally reduced from 512 to 128 using two 3 × 3 convolutional layers [9]. The remaining 74 layers are modularized into six stages. The first stage consists of nine convolutional layers with ReLU activation functions, while the remaining five stages have thirteen convolutional layers with ReLU activation functions. The output of each stage produces belief and affinity maps, which are used for correlating the eight bounding box vertices with their respective centroids. The belief maps contain the nine key points, while the affinity maps contain the vectors from every corner to every centroid. Because DOPE is trained with labeled images, including knowledge of the bounding box dimensions, simple trigonometry can determine the expected angles from the bounding box corners to the corresponding centroids. Combined with an angular threshold, this knowledge enables the affinity maps to successfully pair key points while being able to identify multiple objects at once. The matched key points are then passed to a Perspective-n-Point (PnP) [22] solver, which estimates the 6-DoF pose of the objects relative to the camera used to acquire the images. While it is possible to use fewer stages to increase the speed of inference, more ambiguities are resolved when the feature vector is passed through the network at a deeper level, resulting in better predictions.

### 3.2. Simultaneous Localization and Mapping

There are many viable options when considering SLAM algorithms to pair with object recognition to produce labeled maps. However, due to its wide range of abilities and versatility, RTAB-Map was chosen to be paired with DOPE to generate the 3D maps to be labeled. Because it can be used as both LiDAR and visual-based SLAM, it has the benefits of accurate localization from LiDAR and an RGB point cloud from an RGB-D camera. This provides DOPE with accurate localization while making it easy to check the placement of the resulting labels inside the RGB point cloud. RTAB-Map is designed to perform in real-time under large-scale and long-term operating conditions. It does this using a working memory (WM) that tracks the most frequently observed locations and then transfers everything else to long-term memory (LTM) [23]. After matching observed locations with those in the WM, the LTM is updated to preserve the locations in storage. This process is continuously repeated until the entire map is stored in LTM. This method effectively solves the problem of more processing time being required as the number of stored locations increases, which could cause delays that would render the map obsolete during real-time operation [23,24]. This memory management technique allows the system to function independently of the map being generated; thus, large-scale maps are not an issue and DOPE can maintain a real-time operating constraint. The performance of RTAB-Map makes it stand out when compared with other SLAM algorithms. It offers precise odometry estimation by combining the LiDAR data, cameras, and other exteroceptive sensors through sensor fusion as long as there are enough environmental features to use as a reference [5]. Odometry is further improved by mixing in available proprioceptive sensors to complement the shortcomings of the other sensors. This synergy between the acquired sensor data improves RTAB-Map’s ability to perform loop closure and enables it to provide accurate localization data, which is ideal when passing the coordinates to DOPE for labeling identified objects.

### 3.3. Semantic SLAM Fusion

**Figure 2:**
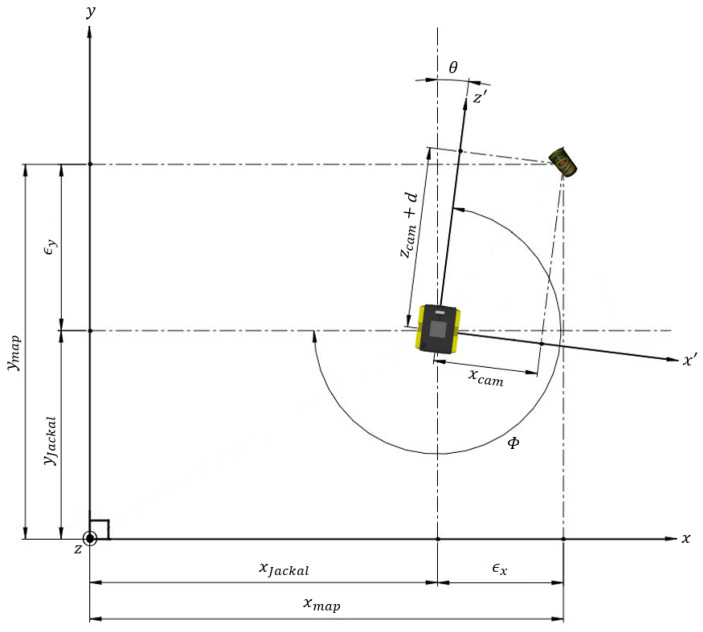
Local camera frame within global map frame.

To combine DOPE and RTAB-Map, it is necessary to perform a coordinate transformation to unify the coordinate system of the local camera frame where objects are identified with the coordinate system of the global frame, where the map is generated. This is achieved using a homogeneous transformation matrix (HTM). An HTM combines a rotation matrix with the translation of an object in a particular frame, then combines the coordinates of an object in a second frame to provide a bridge between the two. An HTM is intended to be used when the object location remains constant while the coordinate system changes. This scenario is visualized in Figure 2. In this case, the HTM is linking the pose of the robot with the relative position of an identified object in the camera frame. First, the angle between the robot in the global frame and the y-axis must be calculated as in Equation (1), then the localization pose of the robot is combined with the 3D coordinates of the object provided by DOPE using an HTM as in Equation (2). The pose of the robot extracted from RTAB-Map is provided by Φ, $x_{Jackal}$, $y_{Jackal}$, and $z_{Jackal}$, where Φ is the yaw angle; as the ground is assumed to be flat, this is the only important angle. The object coordinates from DOPE are represented by $x_{cam}$, $y_{cam}$, and $z_{cam}$, while *d* is the camera offset to the center of the robot that allows the angles to be synchronized. Note that *d* is not a necessary parameter when using a Unified Robot Description Format (URDF) file in ROS, as the transforms between all components on the robot can be defined. While Figure 2 is a demonstration of how a single camera can be used with an HTM, it is possible to use multiple cameras as well. However, θ from Equation (1) would need to be appropriately adjusted to align with the $z^{\prime }$ axis of each camera. Because RTAB-Map and DOPE can run independently and combine their results afterwards, this is a loose-coupled parallel fusion approach. Using a rosbag file, raw data can be collected from a robot passing through a given area. The raw data can either be used by RTAB-Map to perform SLAM and generate the point cloud while recording the localization coordinates separately, or RTAB-Map can be used while gathering the raw data initially, as it is capable of real-time operation. In any case, a rosbag is created that stores the SLAM-generated map. Finally, DOPE can use the SLAM rosbag to label objects found within the map, which afterwards produces a fully annotated 3D map. When using this method the system is easy to implement, the computation cost is low because the algorithms are running independently, and the SLAM algorithm can easily be swapped out if desired. This embedded flexibility is one of the greatest strengths of using loose-coupled parallel fusion for this task.

(1) $$\theta =\frac{3\pi }{2}-\Phi$$

(2) $$\left[\begin{array}{c} x_{map}\\ y_{map}\\ z_{map}\\ 1 \end{array}\right]=\left[\begin{array}{cccc} \cos\theta & \sin\theta & 0 & x_{Jackal}\\ -\sin\theta & \cos\theta & 0 & x_{Jackal}\\ 0 & 0 & -1 & z_{Jackal}\\ 0 & 0 & 0 & 1 \end{array}\right]\left[\begin{array}{c} x_{cam}\\ z_{cam}+d\\ y_{cam}\\ d \end{array}\right]$$

### 3.4. Depth Map Object Recognition

While the original version of DOPE performs well in extremely bright light [9], its ability to recognize objects in darkness is non-existent. By modifying DOPE’s pipeline to accept depth maps as an alternative input to RGB images, it can leverage the ability of infrared cameras used in active triangulation [25] to identify objects based on their shape in absolute darkness, under camouflage, or in other low-visibility conditions. This modification was inspired by the real-world need to identify objects in complete darkness inside floating-lid fuel tanks for an industrial partner.

**Figure 3:**
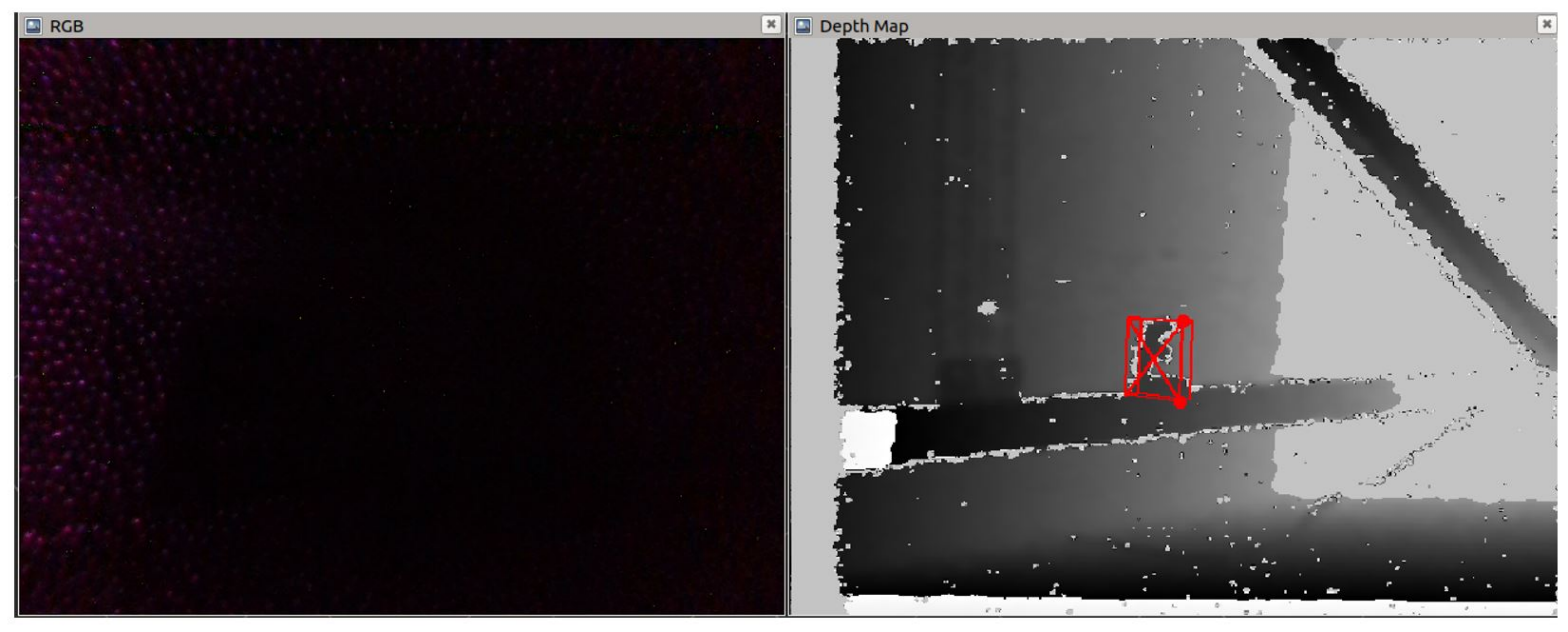
Comparison of RGB (**left**) and depth map (**right**) used as input to DOPE in darkness.

A raw depth map is an inherently noisy image; because it is in essence a compressed point cloud, there are several challenges that need to be overcome in order for it to be an effective input to an object recognition algorithm. An important distinction between a depth map and an RGB image is that the pixel intensities in a depth map represent distance rather than color. This quality can be leveraged to remove most of the noise that would otherwise inhibit object recognition. By removing the high intensities in a depth map, a high-contrast background can be established, making an object’s shape more easily identifiable. Furthermore, by removing the low intensities, artifacts that appear due to the interaction between the two infrared cameras that create the depth map, which appear as shadows, are removed. By removing both the high and low intensities simultaneously, any saturation effects from reflections in the environment are removed. This effectively slices the depth map and leaves a detection window of a size determined by the intensity thresholds that have been replaced with uniform values. It is imperative that the detection window is within the maximum observable range of the algorithm, which for DOPE is useful up to approximately 150 cm. The result of performing this operation can be seen in Figure 3 in a side-by-side comparison in complete darkness, with an RGB image used as input to DOPE on the left and a sliced depth map used as input on the right.

**Figure 4:**
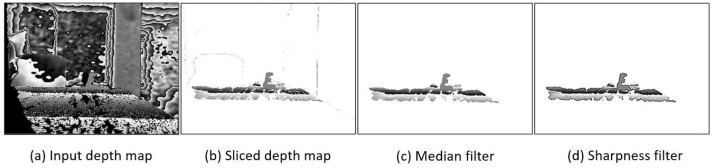
Input depth map and modified depth map after filtering.

While slicing the depth map removes most of the noise and enables object recognition, the image quality of the depth map can be further improved. As seen in Figure 3, after slicing the depth map there is high-frequency noise remaining in the image, visible as small holes or white dots. This high-frequency noise can be removed using a low-pass filter. While there are many filters to choose from, using a median filter is the ideal choice because edges can be preserved, which is important because object recognition is entirely shape-based when using a depth map as input. In this case, five median filters with a 3 × 3 kernel are used to remove the high-frequency noise while preserving the edges. A single sharpness filter is applied to the depth map to further improve the edge quality before the map is passed to the CNN. While edge detection can be built into a CNN [26], a CNN uses a weighted sum of neighboring values, meaning that the effects of a median filter cannot be learned in an end-to-end model. For this reason, median filtering needs to be applied before being passed to the network in order to fully realize the benefits. Figure 4 shows the input depth map and the modified depth map after filtering.

### 3.5. Precision Enhancement

The precision enhancement method, called FPR, leverages the belief map system that DOPE uses to identify the key points of the bounding boxes. As previously mentioned, after each stage a set of belief and affinity maps are used as the output to respectively estimate the pose of the bounding box and connect the points to the centroid. After each stage, the belief and affinity maps are used as input in the following stage. As they progress through the CNN, ambiguities are resolved and confidence in the results increases. However, even after all six stages there may not always be high confidence in the results. If the confidence in the results is high, this represents well-defined points with few ambiguities.

If all the values of the input tensor are added together during a prediction with well-defined points, it is possible to establish a threshold that would be seen during a correct prediction. If, on the other hand, the tensor is added together and the value is much higher than the threshold, this means one of two things: either there are multiple objects being identified, or the confidence in the results is low. When filtering out all predictions that are above the established threshold, only those predictions with high confidence are permitted. This means that precision can be drastically increased while sacrificing recall, as many predictions are ignored. Another side effect of this method is that only one object can be identified at a time, as even if there are two sets of well-defined points this is considered an uncertain detection. However, if the appropriate threshold is used it is possible to maximize precision while maintaining balance, while keeping recall at a level that is ideal for semantic labeling of a 3D map.

### 3.6. Persistent State Observation

An important function that neither RTAB-Map, RViz, nor DOPE has is the ability to store the locations of the labeled objects on the final map. By saving the state of an identified object (such as its position, orientation, mesh marker, bounding box, and time stamp) to an Excel file, it can be stored for later use. This is achieved by storing the information in a data structure converted to Excel format using the Pandas Python library.

Each object stored has a unique ID number. If the same object is found, the state is updated; if a new object is found, a new entry is added. This is a crucial component of the entire system, as otherwise the results of running RTAB-Map and performing object recognition on the point cloud cannot be stored in a final annotated map. After all the objects have been saved in the Excel file, they can be launched simultaneously using a roslaunch file custom-made for this purpose. Alternatively, the point cloud of the map can be loaded alongside the pose markers of the stored objects into a program such as Blender and converted into a single 3D object, allowing it to be used in any other desired software.

**Figure 5:**
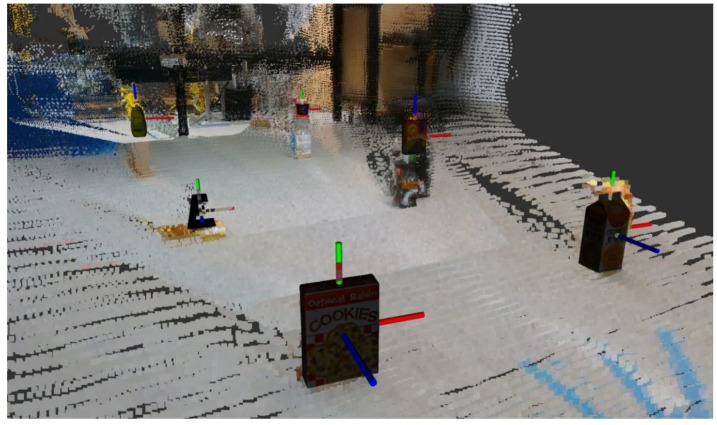
Manually adjusted labeled map with object mesh and pose markers from DOPE fused with RTAB-Map point cloud.

Another benefit of saving the state of all identified objects in an Excel file is that the labels can be manually edited. While it may seem that this is duplicitous or bypassing the point of the system, allowing manual adjustment is a major improvement to what previously existed. While it may not be autonomous, this ability allows the final map to have near-perfect labels, as seen in Figure 5. If the system is fully autonomous, manual adjustment can be ignored, and will have no impact on the rest of the mapping system; this is purely an optional post-processing step for fine-tuning of the labels on the resulting map.

## 4. Methodology

The proposed semantic SLAM system was evaluated through three different experiments. First, DOPE’s ability to provide accurate depth estimation was evaluated while determining the ideal size of the dataset to use for training. The optimal weights found during the first experiment were then used in a second experiment in which the depth map object recognition modification was used to determine the precision, recall, and F-score. These values were quantified as the number of layers in DOPE’s network was reduced from 86 to 21 while observing the resulting increase in frame rate. Finally, after validating FPR it was used in the final experiment, in which the overall performance of the proposed semantic SLAM system was tested. The results when using FPR were compared with the original version as well as with the best detections, which were used as a baseline, and the worst detections, which were to place the range in perspective.

### 4.1. Hardware

Four main pieces of hardware were used to implement the proposed semantic mapping system. Both RTAB-Map and DOPE rely on the use of an RGB-D camera, in the first instance for generating the RGB point cloud and in the second for placing the labels on the objects found within it. Additionally, the depth channel of the RGB-D camera is used as input to DOPE’s modified pipeline for depth map object recognition. The RGB-D camera used for this purpose was an Intel RealSense D435i. Additionally, RTAB-Map uses a Velodyne 32MR LiDAR for determining the localization coordinates that are eventually passed to DOPE for converting object coordinates to the map frame. Clear-Path’s Jackal mobile robot was used as the platform that each sensor was mounted to while navigating through the environment. The Jackal has an onboard computer equipped with an Nvidia RTX 1080 Ti GPU, and uses an Ubuntu 18.04 operating system running ROS Melodic for communication and control. Finally, for training the weights used in DOPE’s CNN and for measuring the frame rate in the second experiment, an Nvidia Titan RTX GPU was used on a dedicated computer using the Ubuntu 18.04 operating system.

### 4.2. Depth Estimation and Dataset Size

The first experiment was designed to evaluate the performance of DOPE’s depth estimation while determining the optimal size of the dataset to use when training a model. This experiment is similar to one performed in [9], although it takes a more practical approach in that it uses a physical grid instead of the average distance metric outlined in [27], which uses incremental tolerances overlayed on a simulated 3D grid. The ground truth in this experiment is obtained by creating a grid on a flat surface that spans from −30 cm to 30 cm horizontally in the *x* direction and 170 cm into the image in the *z* direction, with the origin at the center of the D435i RGB lens. These distances were chosen based on DOPE’s effective range. As the vertical position was held constant throughout the experiment, only the *x* and *z* directions were tested.

To evaluate the accuracy of each model, five different points on the grid were tested for a given dataset. A test object was 3D printed and used as the subject for the test, and its 3D model was used to generate the training images with NDDS. The object had an “E” shape, which was chosen for its distinct shape and ease of printing. When the object was identified, the output coordinates from DOPE were recorded and compared with the ground truth obtained from the grid, then the accuracy was found relative to the origin of the grid.

The models used for experimentation were trained with five different datasets ranging from 50 k to 400 k images, and were trained over 60 epochs with a batch size of 32 to produce five different sets of weights. Each dataset was generated using NDDS and was populated with synthetic RGB, depth maps, and segmentation images. The model was validated with a dataset that was 20% the size of a given dataset which was generated the same way using NDDS. Brightness and contrast were randomized during training, and Gaussian noise was added to the images. A learning rate of 0.0001 was used in each case, along with the ADAM optimizer, which uses adaptive learning rates [28]. Furthermore, an L2 loss function was used to avoid the vanishing gradient problem [9] during training. Several different learning rates and epochs were tested and evaluated based on the average loss, which was ultimately used to determine the selection of these values. The performance of each model is evaluated experimentally in Section 5.1.

### 4.3. Precision Quantification

The second experiment evaluated the effectiveness of FPR using a novel method to quantify the improvement that it offers. This experiment used the depth recognition module, and used weights that were retrained to exclude the RGB images using the 100 k dataset determined to be the optimal size in Section 5.1.

FPR was applied to the sliced and filtered depth maps, and was first compared with the sliced and filtered depth maps on their own for use as the baseline. By reducing the number of stages from six to one, the network was reduced from 86 to 21 layers, meaning that there were twelve different configurations; these are represented as $D_{i,j}$, where *i* represents the number of stages being used and *j* represents whether FPR is enabled (*F*) or not (0). The changes in precision, recall, and F-score and the resulting increase in frame rate were recorded at each stage.

To determine how many successful predictions were made, the number of False Positives (FP) was first determined. To find this number, the same test object from the previous experiment was placed in a static location 80 cm from the D435i camera with a 30 cm detection window; when a correct prediction from DOPE was observed, the coordinates were recorded as the ground truth. During the test, if the output location provided by DOPE was greater than 5 cm in any direction from the ground truth this was considered to indicate a false positive, and a counter was incremented to this effect; the FP value was found after 60 s. The total number of True Positive (TP) predictions was recorded at the same time. Then, the number of Correct Predictions (CP) was found by subtracting FP from TP. Additionally, the average frame rate was recorded. By multiplying the frame rate by 60 s, the total number of frames and the Total Instances (TI) of the object could be found, as it was in every frame and in a static location. Based on this, the number of False Negatives (FN) was found by subtracting CP from TI. The relationships between these values were then used to calculate the precision and recall values with Equations (3) and (4), respectively.

(3) $$Precision\left(P\right)=\frac{CP}{CP+FP}=\frac{CP}{TP}$$

(4) $$Recall\left(R\right)=\frac{CP}{CP+FN}=\frac{CP}{TI}$$

To determine the overall performance of an object recognition algorithm, the balance between precision and recall can be measured with the F-score, as in Equation (5). The F-score is calculated as the harmonic mean between the precision and recall [29]. The harmonic mean is used rather than the arithmetic mean in order to punish extreme values. Because FPR forcibly reduces recall in order to maximize precision, it is expected that the F-score will be impacted accordingly. It is important to maximize precision for this application because false positives have a detrimental impact on the quality of the final map. While it is important to maintain a reasonable recall, we want to ensure that the best possible map is produced in the end; thus, the focus is to maximize precision and not to optimize the F-score.

(5) $$\text{F-score}\left(F\right)=\frac{2PR}{P+R}=\frac{2CP}{2CP+FP+FN}=\frac{2CP}{TP+TI}$$

One downside of the depth map recognition module introduced to DOPE is that false positive predictions are even more prevalent than with RGB images. However, this experiment leverages this weakness and turns it into a strength for the purpose of quantifying FPR. Under the same conditions, using RGB images would result in consistent correct bounding box placement even when only using a single stage. Contrarily, when using a depth map for object recognition, it is more challenging to identify the object, and as such DOPE is often unable to place the bounding box in the correct location. This makes it possible to quantify the improvement that FPR offers. FPR can then be used to offer a similar improvement when in motion and using RGB images, which are cases where false positives are more frequent.

### 4.4. Object Localization

The third and final experiment used the results from the previous two experiments to evaluate the performance of the overall system by combining DOPE, FPR, and RTAB-Map using an HTM. Six different objects were placed in two columns at various heights, creating a path for the Jackal to drive as it labeled the objects and placed them in a final map. The performance metric used in this experiment was the distance between the output from DOPE and the approximate centroids of the objects within the RGB point cloud generated by RTAB-Map. Because the ground truth was extracted from RViz, the precise locations of the objects were not required when physically placing them. To remove any bias, the Jackal passed between the objects in one continuous motion and the last bounding box location was used as the label for the object even if it was a false positive. Each object tested the system’s ability by comparing DOPE’s default settings to those with FPR enabled, which was then compared against the manually selected best labels used as a baseline. Additionally, the worst labels were recorded to provide greater perspective on the available range in which an object could be incorrectly placed, which can highlight the importance of FPR. In addition to the custom-trained object from the previous experiments, DOPE was supplemented by five items from the HOPE dataset [30], which is a collection of toy household objects with pre-trained weights. The objects used in the experiment were orange juice, yogurt, mustard, raisins, and cookies. Similar to the custom-trained object, each set of weights were trained with 120 k images [30] to ensure a fair comparison among all tested objects.

To ensure maximum precision when labeling objects, all six stages were used for every test, as determined by the results in Section 5.2 Furthermore, the threshold was calibrated for FPR before testing; however, it was adjusted through trial and error and was not necessarily optimal.

Each object was tested individually. To guarantee the test was the same for each run, a rosbag file was used to store the sensor data and was replayed each time. A 60-s recording was used for each test, of which only 30 s involved motion from the Jackal and the other 30 s was used to generate the initial map for updating the localization pose before the Jackal started moving.

## 5. Results and Discussion

As described in the previous section, the results from each experiment were successively used in the following experiment, culminating in the final experiment that evaluated the overall performance of the system. Using the ideal dataset size determined in the first experiment, a model for the depth recognition module was trained and used to quantify FPR. Finally, after quantifying the improvement that FPR provided when using depth maps for object recognition, FPR was used with RGB images as the input while the robot was in motion. The output coordinates from DOPE were then unified with RTAB-Map through the use of an HTM, and the resulting distance from the ground truth was used as the performance metric for the overall system.

### 5.1. Depth Estimation and Dataset Size

The results from testing all five points for each of the five different datasets from 50 k to 400 k can be found in Table 1. The difference between the x-axis ground truth $T_{x}$ and the output from DOPE $D_{x}$ is represented by Δx. Similarly, Δz represents the difference between $T_{z}$ and $D_{z}$. Using these values, the distance between the ground truth and DOPE’s output is found with Equation (6), and the accuracy relative to the origin is found with Equation (7). Finally, the average and standard deviation of all five tested points are used to evaluate the performance of a given dataset.

(6) $$Distance=\sqrt{{\left(\Delta x\right)}^{2}+{\left(\Delta z\right)}^{2}}$$

(7) $$Accuracy=\left(1-|\frac{\sqrt{T_{x}^{2}+T_{z}^{2}}-\sqrt{D_{x}^{2}+D_{z}^{2}}}{\sqrt{T_{x}^{2}+T_{z}^{2}}}|\right)$$

From Table 1, the largest source of error is the depth estimation, while the horizontal error is relatively stable across all datasets. This is expected, as the x-coordinate is relatively easy to determine in the frame; it is perpendicular to the camera, while the depth estimation is dependent on DOPE’s estimation of the key points and subsequent PnP output into the image.

The depth estimation along the z-axis has a high degree of variance. The convention of the data is such that a negative number indicates that a result is on the far side of the ground truth and a positive number indicates that a result is towards the origin. The average error in the z-coordinate ranges from 0.65 cm away from the ground truth on the 400 k dataset to 3.98 cm away from the ground truth on the 200 k dataset, and the standard deviation reaches a maximum of 7.45 cm on the 400 k dataset. Even though the 400 k dataset has the smallest average depth estimation error, the large standard deviation suggests that further analysis is required before the best dataset can be selected.

An alternative perspective is shown when observing the average distance from the ground truth in each test. After the 100 k dataset with an average distance of 3.47 cm the average distance from the ground truth begins to rise, ultimately reaching 6.36 cm on the 400 k dataset. This result contradicts the results when observing the average depth estimation error alone. Even though the depth estimation error appears to decrease, the overall accuracy tells a different story as the dataset moves beyond 200 k.

The accuracy describes how close the estimated position of the test object is relative to the origin. The highest accuracy was achieved on the 100 k dataset, reaching a maximum of 96.8%, and steadily decreased to 94.8% on the 400 k dataset. Even though the average Δz is the smallest for the 400 k dataset, suggesting that it should see the best performance, this is not the case. This is confirmed by the average distance from the ground truth, which increases as more images are added to the dataset. This is likely due to overfitting; extrapolating from these data, larger datasets would be expected to decrease the accuracy even further. Though the accuracy on all of the datasets is relatively close, the differences in training time are significant, making this an important consideration when selecting a dataset along with efficient use of time and optimal performance.

This experiment reached a similar conclusion to that found in [9]. Though they found the best results with 300 k images, the improvement was minor over 100 k and 200 k, showing that all three datasets are viable options in practice. While the error along the x-axis was close to negligible, the error along the z-axis suggested that more images lead to better results. However, additional datapoints would likely refute this assertion, as overfitting begins to become apparent on the 400 k dataset and the accuracy starts to drop. Similarly, the average distance from the ground truth suggests that more images do not necessarily result in better prediction quality. This is corroborated by the average accuracy from each test, where the highest accuracy of 64.8% was achieved with the 100 k dataset.

### 5.2. Precision Quantification

**Figure 6:**
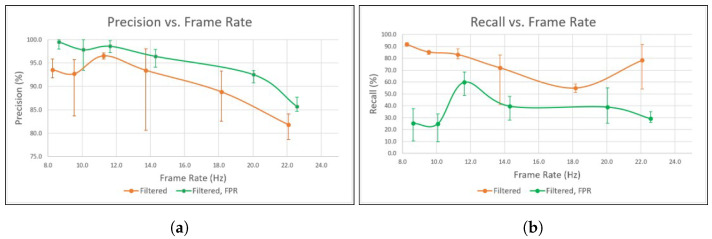
Averaged results from the precision enhancement experiment using twelve configurations, comparing the filtered depth map on its own and with FPR applied against the resulting frame rates, with error bars displaying the maximum and minimum from each test. (**a**) Precision vs. frame rate from stage six (left) to stage one (right). (**b**) Recall vs. frame rate from stage six (left) to stage one (right).

Each of the twelve configurations was tested five times, for a total of sixty tests. The total number of predictions along with the number of false positives and the total number of frames were used to calculate the precision, recall, and F-score using Equations (3)–(5), respectively. The averaged results for the precision and recall are shown in Figure 6, in addition to the highest and lowest values of each test, which are shown as error bars.

From Figure 6a, using FPR clearly offers a significant increase in precision. This allows the depth of the network to be reduced, allowing inference to be realized at a faster rate while maintaining high precision. When using all 86 layers, the configuration with the highest average precision is $D_{6,F}$ at 99.5%, which has a frame rate of 8.6 Hz; when reduced to one stage with 21 layers, $D_{1,F}$ has an average precision of 85.7% and a frame rate of 22.6 Hz. Comparatively, $D_{6,0}$ has an average precision of 93.5% at 8.3 Hz, and drops to 81.8% at 22.1 Hz in $D_{1,0}$. However, the recall of $D_{6,F}$ is 25.2%, which is considerably lower than the 91.5% of $D_{6,0}$. In Figure 6b, $D_{4,F}$ shows a rather substantial increase in recall to 59.7%, which suggests that the FPR thresholds in both $D_{6,F}$ and $D_{5,F}$ were likely too aggressive and could be increased to allow more predictions while maintaining high precision. In contrast, $D_{6,0}$ reached an average recall of 91.5%, which demonstrates how many potential predictions are being ignored when FPR is enabled.

When comparing the variance for each configuration, there is a noticeable deviation when FPR is used. FPR has a much lower variance in precision in almost all cases, as shown in Figure 6a. In the most extreme case, $D_{3,0}$ ranges between 80.6% and 98.1%, while $D_{3,F}$ has a much tighter range between 94.1% and 97.9%. This is due to the nature of FPR, in that it forcibly removes uncertain predictions to increase the ratio of correct to total predictions. This consistency, demonstrated here when using FPR, is ideal when labeling objects on a map.

**Figure 7:**
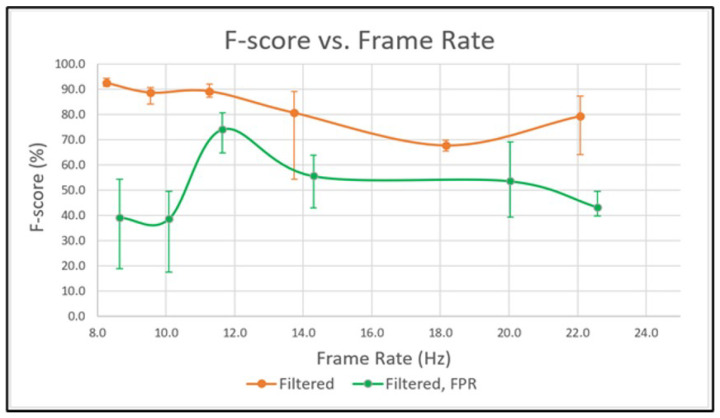
F-score vs. frame rate from stage six (left) to stage one (right).

The overall performance of each configuration can be evaluated with the F-score, as calculated in Equation (5). Figure 7 plots the F-score for each configuration. When compared to Figure 6b, the curve appears to be very similar in shape. This characteristic is due to the F-score being the harmonic mean between precision and recall. When the recall drops sharply, the F-score does as well. The same would be true for precision, however, as it is the value that is to be maximized here it has less of an impact on the shape of the F-score curve. Ideally, one would expect the F-score to be trending downward as the number of layers decreases; however, this does not appear to be the case during this test. Again, this is confirmation that the threshold used in $D_{6,F}$ and $D_{5,F}$ was too stringent and should be increased. This could potentially be improved by using an automated thresholding system to remove any human error that is introduced when manually selecting the threshold. However, even with this inconsistency the experiment proves that adding FPR to DOPE’s pipeline offers a measurable improvement that can be applied to the mapping system to produce a better final product. The superior precision of FPR is ideal for label placement, even though recall is reduced.

### 5.3. Object Localization

The results from the object localization experiment can be seen in Table 2. All six stages were used to ensure maximum precision. The magnitude of the difference between DOPE’s output and the ground truth obtained from RViz is isolated in rows for each of the three-dimensional coordinates. This helps to highlight the largest sources of error. Additionally, the total distance of the object from the ground truth, calculated by finding the Euclidean norm, is found using Equation (8) and shown in row one. Furthermore, an additional row that indicates whether the most recent detection was a false positive or not is recorded in the FP row. Finally, the average difference of each coordinate along with the average distance from the ground truth is displayed to help evaluate the overall performance of each method. The comparison here is between the original version of DOPE after applying the HTM, DOPE with enhanced precision using FPR, and the best and worst detections, which were manually selected during testing and are included to provide more perspective on how the system can perform.

(8) $$l=\sqrt{{\left(\Delta x\right)}^{2}+{\left(\Delta y\right)}^{2}{\left(\Delta z\right)}^{2}}$$

From Table 2, it can be seen that FPR clearly offers a considerable improvement over the original version of DOPE. With FPR there was only one false positive out of the six objects, while the original version had four. This results in a dramatic effect on the average distance between both methods; FPR had an average distance of 30.5 cm from the ground truth, while the original version had an average distance of 79.8 cm. Similarly, the average distance for the best detections was only 12.3 cm, while the average distance for the worst detections was 184.4 cm. When comparing the results from Kimera, which had an average distance of 35 to 48 cm [12], and the probabilistic data association method shown in [13], which had an average distance of 25 cm, the 30.5 cm result for the proposed system using FPR is very comparable. If the result for the cookies, which was the only false positive detected with FPR, were to be excluded, the results would be even better, with an average distance of only 19.1 cm. The results from the cookies for the original version of DOPE and the version with FPR enabled are noticeably present among the worst detections, suggesting that this was a challenging object for the methods using the pre-trained weights as well.

One noticeable observation congruent across all methods is that the average error in the z-coordinate does not have much variance; the best possible detections have an average error in the z-coordinate of 8.3 cm, while the worst possible detections have an error of 10.9 cm, all of which were false positives. This result suggests that DOPE struggles with vertical placement. Unlike its horizontal range, DOPE’s vertical range is limited by the edges of the camera frame, and the variance in the z-coordinate is noticeably inhibited. However, even with this impediment it remains the case that using FPR with DOPE is comparable to other semantic SLAM methods on the same metric.

Overall, the system has two main limitations. First, it lacks an adaptive threshold to tune the false positive rate (FPR) in order to increase the recall while maximizing the precision. An adaptive threshold would allow the system to achieve the best trade-off between correctly identifying true positives and minimizing false positives. Second, the range of the depth maps that the system can process is limited to 110 cm. This means that any objects beyond this range may not be accurately detected, or even detected at all. This limitation can impact the overall effectiveness of the system, particularly in applications where it is necessary to detect objects at greater distances.

## 6. Conclusions and Future Work

This research integrated DOPE with RTAB-Map through loose-coupled parallel fusion, using ROS to create a fully functional high-precision semantic SLAM system that can generate and annotate a 3D map. First, the precision was enhanced by leveraging DOPE’s belief map system to filter out uncertain key points when predicting bounding box locations. DOPE and RTAB-Map were fused together using a homogeneous transformation matrix to unify the local and global coordinate systems to generate the map and correctly position the labels within it. This solution outperforms existing alternatives by promoting an increase in DOPE’s inference speed using the precision enhancement method, allowing the number of layers in the network to be reduced to facilitate object recognition at real-time frame rates. Second, a new perspective for object recognition was added to DOPE’s pipeline in the form of infrared-generated depth maps for use in low-visibility conditions, which further made it possible to quantify the proposed precision enhancement method. Third, the optimal size of the dataset for use in training was determined experimentally. Finally, a method for storing and loading the state of each object was implemented to alleviate the shortcomings of both RViz and DOPE in this area.

The proposed method is designed to enhance the labeling of objects and the generation of 3D maps in dynamic environments by combining DOPE with RTAB-Map. While a quantifiable comparison of the proposed method with all the existing approaches must be subjective in that each technique may be more powerful and practical in certain scenarios and not directly comparable to others, the experiments demonstrated in this paper show that the modifications applied to the algorithms lead to improvements in accuracy and precision when annotating 3D maps in dynamic environments. However, this method’s unique feature of identifying objects in darkness comes with the trade-off of reduced range and reliability due to noisy single-channel depth maps.

In conclusion, the proposed solution allows for autonomous mapping, and additionally makes it possible to manually adjust the location of the labels. Near-perfect labels can be produced for every map in cases where the system does not need to be autonomous. The combination of these elements produces a viable semantic SLAM system that can generate 3D annotated maps that are comparable with and potentially able to outperform existing methods.

Future work that could further improve the performance of the proposed system includes an adaptive thresholding system to automatically tune the threshold needed for FPR, which would allow recall to be increased while maximizing precision. Additionally, the range of depth map object recognition could be increased by sliding the detection window through the entire available range rather than keeping it at a fixed location. In its current state, depth map object recognition has significant limitations; however, it has great potential for certain applications.

## Figures and Tables

**Table 1:** Results from the dataset size and depth estimation experiment; D. is distance in cm and Acc. is accuracy as a percentage.

	50 K				100 K				200 K				300 K				400 K			
	Δx	Δz	**D.**	**Acc**	Δx	Δz	**D.**	**Acc**	Δx	Δz	**D.**	**Acc**	Δx	Δz	**D.**	**Acc**	Δx	Δz	**D.**	**Acc**
P1	0.2	−4.7	4.7	93.3	0.1	−4.6	4.6	93.4	−0.2	−6.1	6.1	91.2	−0.1	−5.7	5.7	91.8	−0.2	−5.5	5.5	92.2
P2	1.4	−4.6	4.8	95.4	1.9	−3.2	3.7	96.5	0.2	−1.0	1.0	99.0	−0.2	−2.2	2.2	98.0	0.2	0.8	0.9	99.3
P3	0.2	0.4	0.5	99.6	0.3	−0.5	0.6	99.7	−1.3	−5.7	5.8	95.3	−1.0	−5.5	5.6	95.5	−1.3	−7.1	7.3	94.1
P4	0.0	−4.9	4.9	96.5	0.1	−4.9	4.9	96.5	0.1	−5.5	5.5	96.0	0.1	−4.7	4.7	96.6	0.2	−4.8	4.8	96.6
P5	−0.8	6.8	6.8	95.8	−1.1	3.4	3.5	97.9	0.1	−1.6	1.6	99.1	−0.9	3.8	3.9	97.6	−2.4	13.3	13.5	91.7
Avg.	0.2	−1.4	4.3	96.1	0.3	−2.0	3.5	96.8	−0.2	−3.9	4.0	96.1	−0.4	−2.9	4.4	95.9	−0.7	−0.7	6.4	94.8
Std.	0.7	4.6	2.1	2.1	1.0	3.1	1.6	2.1	0.6	2.2	2.3	2.9	0.4	3.6	1.3	2.2	1.0	7.5	4.1	2.8

**Table 2:** Object localization experimental results using the average Euclidean norm as the metric for the overall performance of the system. Bold values represent the best results. (N) denotes No, and (Y) denotes Yes.

	Original							FPR						
	**Orange Juice**	**Yogurt**	**Mustard**	**Raisins**	**Cookies**	**Custom**	**Average**	**Orange Juice**	**Yogurt**	**Mustard**	**Raisins**	**Cookies**	**Custom**	**Average**
Δx	15.1	53.2	22.4	66.5	87.3	141.5	**64.3**	9.8	24.4	15.3	7.1	87.3	19.2	**27.2**
Δy	7.4	63.7	4.0	73.1	7.6	79.2	**39.2**	5.2	16.4	0.4	1.3	7.6	4.1	**5.8**
Δz	9.6	8.8	10.6	6.8	2.1	26.8	**10.8**	5.4	9.7	9.4	10.6	2.1	8.3	**7.6**
*l*	19.4	83.5	25.1	99.1	87.6	164.4	**79.8**	12.4	31.0	18.0	12.8	87.6	21.3	**30.5**
FP	N	Y	N	Y	Y	Y		N	N	N	N	Y	N	
	**Best**							**Worst**						
	**Orange Juice**	**Yogurt**	**Mustard**	**Raisins**	**Cookies**	**Custom**	**Average**	**Orange Juice**	**Yogurt**	**Mustard**	**Raisins**	**Cookies**	**Custom**	**Average**
Δx	4.0	9.8	15.1	4.2	2.7	5.8	**6.9**	111.6	214.5	214.3	171.1	87.3	141.5	**156.7**
Δy	1.2	11.8	0.3	1.9	3.8	1.0	**3.3**	101.5	161.8	56.7	129.3	7.6	79.2	**89.4**
Δz	6.1	5.9	9.5	9.9	9.2	9.3	**8.3**	6.7	14.4	9.1	6.4	2.1	26.8	**10.9**
*l*	7.4	16.4	17.8	10.9	10.3	11.0	**12.3**	151.0	269.1	221.9	214.5	87.6	164.4	**184.4**
FP	N	N	N	N	N	N		Y	Y	Y	Y	Y	Y	

## Data Availability

Data sharing not applicable.

## References

[1] Shan T., Englot B. Lego-loam: Lightweight and ground-optimized lidar odometry and mapping on variable terrain. Proceedings of the 2018 IEEE/RSJ International Conference on Intelligent Robots and Systems (IROS).

[2] Koide K., Miura J., Menegatti E. (2019). A portable three-dimensional LIDAR-based system for long-term and wide-area people behavior measurement. Int. J. Adv. Robot. Syst..

[3] Rosinol A., Violette A., Abate M., Hughes N., Chang Y., Shi J., Gupta A., Carlone L. (2021). Kimera: From SLAM to spatial perception with 3D dynamic scene graphs. Int. J. Robot. Res..

[4] Mur-Artal R., Montiel J.M.M., Tardos J.D. (2015). ORB-SLAM: A versatile and accurate monocular SLAM system. IEEE Trans. Robot..

[5] Labbé M., Michaud F. (2019). RTAB-Map as an open-source lidar and visual simultaneous localization and mapping library for large-scale and long-term online operation. J. Field Robot..

[6] Chen S., Zhou B., Jiang C., Xue W., Li Q. (2021). A lidar/visual slam backend with loop closure detection and graph optimization. Remote Sens..

[7] Li Y., Ibanez-Guzman J. (2020). Lidar for autonomous driving: The principles, challenges, and trends for automotive lidar and perception systems. IEEE Signal Process. Mag..

[8] Yu J., Choi H. (2021). YOLO MDE: Object detection with monocular depth estimation. Electronics.

[9] Tremblay J., To T., Sundaralingam B., Xiang Y., Fox D., Birchfield S. (2018). Deep object pose estimation for semantic robotic grasping of household objects. arXiv.

[10] Kasaei S.H., Tomé A.M., Lopes L.S., Oliveira M. (2016). GOOD: A global orthographic object descriptor for 3D object recognition and manipulation. Pattern Recognit. Lett..

[11] Chen Z., Zhang W., Li F., Shi Y., Wang Y., Nie F., Zhu C., Huang Q. A research on the fusion of semantic segment network and SLAM. Proceedings of the 2019 IEEE International Conference on Advanced Robotics and Its Social Impacts (ARSO).

[12] Rosinol A., Abate M., Chang Y., Carlone L. Kimera: An open-source library for real-time metric-semantic localization and mapping. Proceedings of the 2020 IEEE International Conference on Robotics and Automation (ICRA).

[13] Bowman S.L., Atanasov N., Daniilidis K., Pappas G.J. Probabilistic data association for semantic slam. Proceedings of the 2017 IEEE International Conference on Robotics and Automation (ICRA).

[14] McCormac J., Handa A., Davison A., Leutenegger S. Semanticfusion: Dense 3d semantic mapping with convolutional neural networks. Proceedings of the 2017 IEEE International Conference on Robotics and Automation (ICRA).

[15] Li C., Xiao H., Tateno K., Tombari F., Navab N., Hager G.D. Incremental scene understanding on dense SLAM. Proceedings of the 2016 IEEE/RSJ International Conference on Intelligent Robots and Systems (IROS).

[16] Zheng L., Zhu C., Zhang J., Zhao H., Huang H., Niessner M., Xu K. (2019). Active scene understanding via online semantic reconstruction. Comput. Graph. Forum.

[17] Chen X., Milioto A., Palazzolo E., Giguere P., Behley J., Stachniss C. Suma++: Efficient lidar-based semantic slam. Proceedings of the 2019 IEEE/RSJ International Conference on Intelligent Robots and Systems (IROS).

[18] To T., Tremblay J., McKay D., Yamaguchi Y., Leung K., Balanon A., Cheng J., Hodge W., Birchfield S. NDDS: NVIDIA deep learning dataset synthesizer. Proceedings of the CVPR 2018 Workshop on Real World Challenges and New Benchmarks for Deep Learning in Robotic Vision, Salt Lake City.

[19] Tobin J., Fong R., Ray A., Schneider J., Zaremba W., Abbeel P. Domain randomization for transferring deep neural networks from simulation to the real world. Proceedings of the 2017 IEEE/RSJ International Conference on Intelligent Robots and Systems (IROS).

[20] Reway F., Hoffmann A., Wachtel D., Huber W., Knoll A., Ribeiro E. Test method for measuring the simulation-to-reality gap of camera-based object detection algorithms for autonomous driving. Proceedings of the 2020 IEEE Intelligent Vehicles Symposium (IV).

[21] Simonyan K., Zisserman A. (2014). Very deep convolutional networks for large-scale image recognition. arXiv.

[22] Mansour M., Davidson P., Stepanov O., Piché R. (2021). Towards Semantic SLAM: 3D Position and Velocity Estimation by Fusing Image Semantic Information with Camera Motion Parameters for Traffic Scene Analysis. Remote Sens..

[23] Labbe M., Michaud F. (2013). Appearance-based loop closure detection for online large-scale and long-term operation. IEEE Trans. Robot..

[24] Labbé M., Michaud F. Memory management for real-time appearance-based loop closure detection. Proceedings of the 2011 IEEE/RSJ International Conference on Intelligent Robots and Systems.

[25] Foix S., Alenya G., Torras C. (2011). Lock-in time-of-flight (ToF) cameras: A survey. IEEE Sens. J..

[26] Liu Y., Cheng M.M., Hu X., Wang K., Bai X. Richer convolutional features for edge detection. Proceedings of the IEEE Conference on Computer Vision and Pattern Recognition.

[27] Hinterstoisser S., Lepetit V., Ilic S., Holzer S., Bradski G., Konolige K., Navab N. (2013). Model based training, detection and pose estimation of texture-less 3d objects in heavily cluttered scenes. Proceedings of the Computer Vision–ACCV 2012: 11th Asian Conference on Computer Vision.

[28] Kingma D.P., Ba J. (2014). Adam: A method for stochastic optimization. arXiv.

[29] Guns R., Lioma C., Larsen B. (2012). The tipping point: F-score as a function of the number of retrieved items. Inf. Process. Manag..

[30] Tremblay J., Tyree S., Mosier T., Birchfield S. Indirect object-to-robot pose estimation from an external monocular rgb camera. Proceedings of the 2020 IEEE/RSJ International Conference on Intelligent Robots and Systems (IROS).

